# Reduction of oil uptake in vacuum fried *Pleurotus eryngii* chips via ultrasound assisted pretreatment

**DOI:** 10.3389/fnut.2022.1037652

**Published:** 2022-11-09

**Authors:** Aiqing Ren, Zhenzhen Cao, Xiaoxian Tang, Zhenhua Duan, Xu Duan, Xiangyong Meng

**Affiliations:** ^1^College of Food and Bioengineering, Hezhou University, Hezhou, China; ^2^College of Food and Bioengineering, Henan University of Science and Technology, Luoyang, China; ^3^College of Life Sciences, Anhui Normal University, Wuhu, China

**Keywords:** *Pleurotus eryngii*, ultrasound, vacuum frying, pore structure, oil uptake

## Abstract

The reduction of oil uptake in vacuum-fried *Pleurotus eryngii* chips by ultrasound assisted pretreatment was investigated regarding the pore structure changes. Pore structure of *P. eryngii* chips with four pretreatments, such as blanching, blanching + osmosis, blanching + ultrasound and blanching + ultrasound assisted osmosis was determined by mercury intrusion porosimetry (MIP) and scanning electron microscopy (SEM). In addition, the quality parameters of vacuum-fried *P. eryngii* chips such as hardness, rehydration ratio, reducing sugar, protein and oil content were also measured. The results showed that the oil absorption of vacuum fried *P. eryngii* chips was affected by the porous structure. The oil content of vacuum fried *P. eryngii* chips was significantly and positively correlated with the pores with diameters above 50, 5–50, and 0.5–5 μm in the samples both before and after vacuum frying, while negatively correlated with the pores with diameters below 0.5 μm. Ultrasound pretreatment changed the microporous structure of *P. eryngii* chips, effectively hindering the oil absorption of samples. In particular, ultrasound assisted osmosis pretreatment induced the formation of more micropores. It was concluded that blanching + ultrasound assisted osmosis pretreatment is a promising method to reduce oil absorption and improve the quality of vacuum fried foods.

## Introduction

*Pleurotus eryngii* is considered as a delicious and healthy food material, which is rich in protein, polysaccharides, essential amino acids, vitamins and other nutrients (1). Many studies have shown that it has a variety of biological activities such as anti-oxidation, anti-tumor, immune boosting, regulation of intestinal microorganisms and other health care functions (2, 3). However, *P. eryngii* has a moisture content of 89.8%, and a high respiration rate and is rich in nutrients. Therefore, it is susceptible to spoilage due to the microbial reproduction. The short shelf-life of *P. eryngii* mushrooms hinders its exploitation. It is necessary to find a processing technology that can extend the shelf-life of mushroom and produce a snack food with a rich flavor and nutritional value.

Atmospheric frying involves the immersion of foods in edible oil at a temperature above the boiling point of water to make the surface temperature of food rapidly rise (4). Subsequently, a dry layer is quickly formed on the surface of the material, then the water vaporization layer gradually moved inward and finally the food is completely dried and cooked (5). As a consequence, water and other soluble materials are transferred from the food being fried as the oil penetrates the product, resulting in high levels of oil in the food. Furthermore, some adverse reaction products, such as acrylamide may be generated.

Finding a frying technology that could reduce energy consumption and oil uptake and decrease the formation of harmful substances in fried products has become a research hotspot. Some of the innovative frying technologies have emerged, such as pressure frying, microwave frying, or vacuum frying. Vacuum frying is a process carried out under atmospheric pressures (below 100 kPa) leading to decrease the boiling point of water, which makes it possible to considerably decrease the frying temperature (6). Due to the lower frying temperature, vacuum frying preserves the nutritional value and color of the fried product better and keeps the lower oil uptake compared to atmospheric frying. Another approach to reduce oil uptake is the use of different pretreatments prior to frying, such as coating (7), osmotic dehydration and preliminary drying. The reason for the reduction in oil absorption is thought to be related to the increased solids content of the raw material and changes in the microstructure of the material, which favors a reduction in the surface area of the sample in contact with the oil (8). However, it takes a long time for osmosis pretreatment, while ultrasound assisted osmosis pretreatment can effectively shorten the osmosis time and the subsequent drying time, and reduce the energy consumption.

In recent years, ultrasound has attracted widespread attention due to its numerous advantages over traditional methods. It has been widely used in the food processing, such as extraction (9), freezing (10), drying (11, 12), thawing (13), fermentation (14), enzymolysis of protein (15), modification of starch (16), inactivation of enzymes (17), as well as cleaning (18). As a non-thermal processing method, ultrasound pretreatment before frying can improve the crispness of fried food, reduce the loss of nutrients. Piyalungka et al. (19) reported that ultrasound-assisted osmosis pretreatment could improve the osmotic dehydration efficiency of maltodextrin-immersed pumpkin, it also improved the color, texture and carotenoid content of vacuum-fried pumpkin crisps, and reduced the oil content of the products. Although ultrasound pretreatment can reduce the oil content of vacuum-fried crisps, the mechanism of the effect of ultrasound and ultrasound assisted osmosis on the oil absorption of vacuum-fried crisps was not clear (20, 21). Zhang et al. (22, 23) found that ultrasound pretreatment altered the pore structure of potato, which was thought to be related to the oil absorption of fried potato chips. The mechanism of the effect of ultrasound pretreatment on the oil absorption during vacuum frying might be revealed from the differences of the pore structure and distribution of the raw materials.

At present, there is little literature about the effect of ultrasound and ultrasound assisted osmosis on the absorption of oil in vacuum fried fruit and vegetable chips. The purpose of this study was to investigate the effect of the pore characteristics on the oil absorption of vacuum fried *P. eryngii* chips by analyzing the pore characteristics of ultrasound pretreatment and ultrasound assisted osmosis, which is expected to provide a valuable basic theory for controlling the oil content of vacuum fried fruit and vegetable chips.

## Materials and methods

### Pretreatments

Fresh *P. eryngii* without mildew and mechanical damage was selected, washed and cut into 40 mm × 12 mm × 6 mm slices. The chemical composition of fresh *P. eryngii* was determined as shown in Table 1, the indexes were indicated as dry basis. Before frying, the slices were processed using four pretreatments (19, 24, 25): (1) Blanching: the *P. eryngii* slices were blanched in the boiling water at 100°C for 3 min; (2) Blanching + osmosis: the *P. eryngii* slices were blanched in the boiling water at 100°C for 3 min, and then immersed in 40% maltodextrin solution at 30°C for 30 min (the ratio of material to liquid was 1:10); (3) Blanching + ultrasound: the *P. eryngii* slices were blanched in the boiling water at 100°C for 3 min, and then subjected to ultrasound pretreatment (JY92-IIN, Ningbo Xinzhi Biotechnology Co., Ltd., Ningbo, China. 25 kHz, 300 W, pulse time 10 s, interval 2 s) at 30°C for 30 min with samples immersing in distilled water (the ratio of material to liquid was 1:10); (4) Blanching + ultrasound assisted osmosis: after blanching in the boiling water at 100°C for 3 min, the *P. eryngii* slices were immersed in 40% maltodextrin solution (the ratio of material to liquid was 1:10) and subjected to the ultrasound pretreatment at 30°C (25 kHz, 300 W, pulse time 10 s, interval 2 s) for 30 min.

**TABLE 1:** Quality analysis of *Pleurotus eryngii* chips.

	Hardness (g)	Rehydration ratio (g/g)	Reducing sugar (g/100 g)	Protein (g/100 g)	Moisture content (g/100 g)	Oil content (g/100 g)
Fresh	–	–	2.86 ± 0.22^a^	19.35 ± 0.25^a^	880.39 ± 17.65^a^	2.53 ± 0.53^e^
Blanching	–	–	2.05 ± 0.21^b^	18.35 ± 0.18^b^	857.19 ± 12.78^b^	2.05 ± 0.62^e^
Blanching + Osmosis	–	–	1.94 ± 0.19^b^	17.85 ± 0.15^b^	805.15 ± 15.21^c^	2.35 ± 0.54^e^
Blanching + Ultrasound	–	–	1.95 ± 0.25^b^	17.92 ± 0.19^b^	832.46 ± 21.47^b^	2.13 ± 0.55^e^
Blanching + Ultrasound + Osmosis	–	–	1.94 ± 0.18^b^	18.05 ± 0.22^b^	795.36 ± 19.45^c^	2.16 ± 0.53^e^
Blanching (Fried)	842.21 ± 8.51^a^	2.99 ± 0.12^d^	1.95 ± 0.18^b^	18.19 ± 0.12^b^	4.93 ± 0.48^d^	20.54 ± 0.85^a^
Blanching + Osmosis (Fried)	597.18 ± 3.44^b^	3.08 ± 0.14^c^	1.83 ± 0.25^b^	17.83 ± 0.25^b^	4.99 ± 0.45^d^	12.15 ± 0.55^b^
Blanching + Ultrasound (Fried)	484.68 ± 4.24^c^	3.54 ± 0.09^b^	1.88 ± 0.23^b^	17.95 ± 0.24^b^	4.55 ± 0.45^d^	10.53 ± 0.61^c^
Blanching + Ultrasound + Osmosis (Fried)	469.78 ± 1.53^d^	3.63 ± 0.17^a^	1.84 ± 0.27^b^	17.85 ± 0.22^b^	4.85 ± 0.49^d^	8.32 ± 0.65^d^

Values are displayed as mean ± standard deviation. Different letters in the same column indicate significant differences (*P* < 0.05).

### Frying process

The frying process was performed in a vacuum fryer (VF-40C, Zhongshan Weijia Vacuum Machinery Factory, Zhongshan, China). About 40 L of soybean oil (Yihai Kerry, Shanghai, China) was preheated to 90 ± 1°C, and then 300 g of *P. eryngii* slices were fried at 10 kPa for 14 min. After frying, deoiling was performed under vacuum conditions at the speed of 300 r/min for 3 min (25).

### Examination of pore properties

The fried samples were immersed in petroleum ether for 10 min to remove the oil, and then dehydrated by a vacuum freeze dryer (Labconco, Kansas City, MO, USA). The conditions of vacuum freeze drying were that the cold trap temperature of −80°C and a pressure of 5 Pa. Pores with an equivalent diameters of 0.005–350 μm were determined by mercury intrusion porosimetry (MIP) (Poremaster GT-60, Quantachrome Instruments, Boynton Beach, FL, USA) according to the method of Zhang et al. (23).

### Microstructure evaluation

The dehydrated *P. eryngii* chips were cut into slices with thickness of 2 mm and coated with a thin gold layer. The morphology was observed with a scanning electron microscopy (SEM, S4800, Japan, Hitachi High-Tech Group, Tokyo, Japan) under the condition of 5 kV acceleration voltage and vacuum (26).

### Examination of oil distribution

The oil distribution of fried *P. eryngii* chips was determined by LF-NMR (NMI20, Niumag Electronic Technology Co., Ltd., Shanghai, China) as described by Zhang et al. (23) with some modifications. One piece of *P. eryngii* chips was placed in a nuclear magnetic coil (diameter of 12 mm), and the temperature was maintained at 32°C. The signal of proton decay was measured using Carr-Purcell-Meiboom-Gill sequence with the following parameters: the number of slices was 3, slice width was 5.0 mm, repetition time was 500 ms; time echo was 20 ms and number of averages was 4.

### Examination of moisture, protein and oil content

According to the methods described by AOAC with minor modifications (27), the content of moisture, protein and oil was measured and weight as g/g dry basis (db) of *P. eryngii* chips.

### Texture analysis

A texture analyzer (TAXT2i, Stable Micro System Co., Ltd., Surrey, UK) equipped with a cylindrical probe with a diameter of 2.5 mm was used to measure the texture of fried *P. eryngii* chips. The operating conditions were target distance of 5 mm, pre-test speed of 1 mm/s, test speed of 1 mm/s, post-test speed of 10 mm/s. The puncture test was repeated three times (28).

### Determination of rehydration rate

According to the methods described by Yang, Li and Hu (1) with modifications, about 5 g of the sample was accurately weighed and its weight (W0) was recorded. Then the sample was immersed in distilled water for 30 min at 25°C and weighed again (W1). The rehydration ratio RR = (W1 – W0)/W0.

### Determination of reducing sugar content

According to the methods described by Li et al. (29), reducing sugar content was determined by colorimetric method of 3, 5-dinitrosalicylic acid.

### Determination of color

A chromaticity instrument (CR-400, Konica Minolta Sensing, Inc., Osaka, Japan) was used to determine the color of vacuum fried *P. eryngii* chips. The L* represents brightness (0 = black, 100 = white), a* represents red index (-a* is green, + a* is red), b* represents yellow index (-b* is blue, + b* is yellow), △*E* = [(L* - *L*_*o*_)^2^ + (a* - *a_*o*_*)^2^ + (△b* - *b_*o*_*)^2^]^1/2^, where *L*_*o*_, *a*_*o*_, and *b*_*o*_ referred to the color reading of fresh samples, which was used as control. Each sample was determined three times.

### Statistical analysis

All experiments and determinations were repeated three times. Then the results were analyzed by SPSS (SPSS v19.0, IBM, Armonk, NY, USA) and Origin (Origin Lab Corporation, Northampton, MA, USA), and expressed as means ± standard deviations. The significant difference among samples was performed statistically by one-way analysis of variance (ANOVA) with Duncan’s *t*-test at a significance level of *P* < 0.05.

## Results and discussion

### Pore structure

**FIGURE 1:**
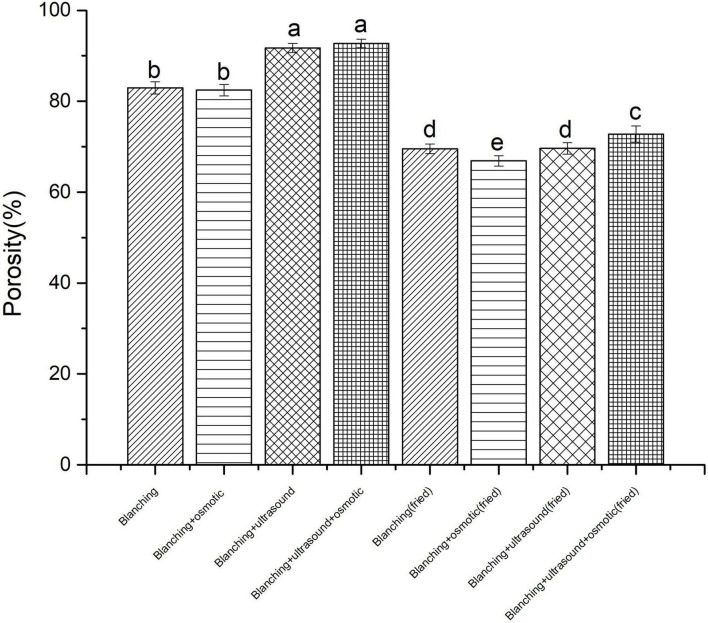
Porosity of *Pleurotus eryngii* chips. Different superscript letters in the graph indicate significant differences between means (*P* < 0.05).

The porosity of *P. eryngii* chips after different pretreatments and vacuum frying measured by MIP were shown in Figure 1. The results showed that the porosity of *P. eryngii* slices pretreated with blanching + ultrasound and blanching + ultrasound assisted osmosis were 91.7 and 92.7%, respectively, which were higher than that of samples treated with blanching (82.94%) and blanching + osmosis (82.44%). The porosity of *P. eryngii* slices with ultrasound pretreatment were greater than that of without ultrasound treated samples. This was consistent with the result reported by Zhang et al. (22), it may be due to the rapid alternating contraction and expansion of ultrasound waves resulting in the formation microchannels.

After vacuum frying, the porosity of *P. eryngii* chips decreased. The order of porosity for fried *P. eryngii* chips treated with different pretreatments was as follows: blanching + ultrasound + osmosis (72.67%) > blanching + ultrasound (69.63%) > blanching (69.52%) > blanching + osmosis (66.88%). There were many studies on the change in porosity of foods during frying. It was reported that the porosity of potato increased after frying (30). The increase in porosity was probably attributed to the disruption of pore structure and the expansion of vapors (31). The different findings in porosity were probably due to that soxhlet extraction and vacuum freeze-drying methods were carried out to remove the oil and water of *P. eryngii* chips before the determination of porosity by mercury intrusion method, resulting in larger porosity because of high moisture content of *P. eryngii* chips before frying. Moreover, Kassama et al. reported the cumulative pore volume and the porosity of chicken meat decreased in 360 s of frying (32, 33). Apart from that, the pore microstructure of vacuum fried and atmospheric fried products was different (34).

**FIGURE 2:**
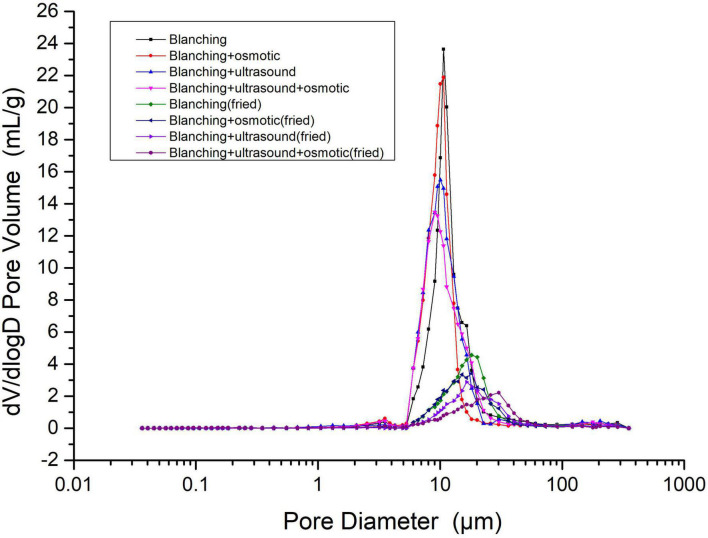
Pore size distribution of *Pleurotus eryngii* chips.

The pore structure of the *P. eryngii* chips treated by different pretreatments was determined by MIP as shown in Figure 2. According to the pore size distribution characteristics, the pore size of *P.* slices could be divided into four regions: above 50, 5–50, 0.5–5 μm and below 0.5 μm. The pore size distribution of *P. eryngii* slices and vacuum-fried *P. eryngii* chips showed a single peak at 5–50 μm, indicating that the pore size distribution of the samples was relatively uniform. The most probable pore diameter of *P. eryngii* slices was 10 μm before frying, but it increased to 20–30 μm after vacuum frying. Zhang et al. also found that the most probable pore diameter of potato chips was 10 μm, and it increased to 50 μm after atmospheric frying (23). The main distribution area of the pore diameter of vacuum fried samples in this study was different from that of atmospheric fried potato chips. Liu et al. (35) found that more than 90% of potato chips had pore size distributions in the range of 10–250 μm. However, Yang et al. (36) reported that the pore size distribution of three types of potato chips, i.e., flat potato chips, batch cooked potato chips, and ridged potato chips, was mainly in 300–800 μm, which differed from the experimental results of Liu et al. (35). Furthermore, the pores volume and pore distribution of the three potato chips was also different. Different frying methods, frying time and material structure of fried samples resulted in various pore distribution (23). The difference in pore size distribution between *P. eryngii* chips in this experiment and the fried potato chips reported in references might be caused by different raw materials or differences between vacuum frying process and atmospheric pressure frying.

**FIGURE 3:**
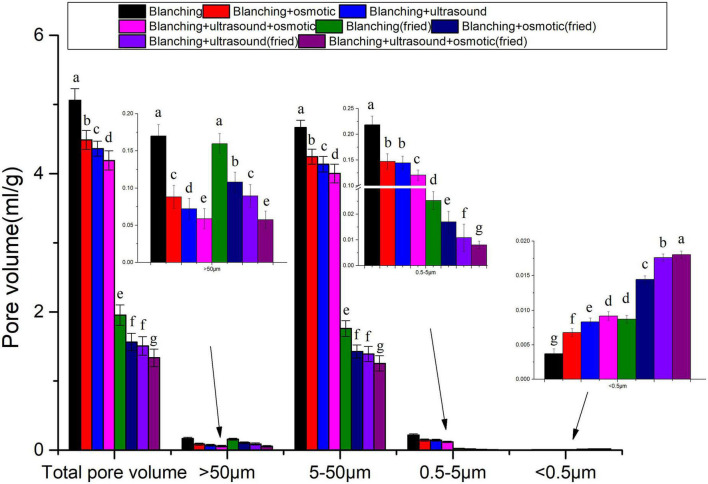
Pore volume distribution of *Pleurotus eryngii* chips. Different superscript letters in the graph indicate significant differences between means (*P* < 0.05).

Figure 3 showed the total pore number (volume) distribution of *P. eryngii* slices. Before vacuum frying, the total pore volume and pore volumes with a diameter of 5–50, 0.5–5 μm and above 50 μm of *P. eryngii* slices pretreated with different methods showed the same trend and followed the following order: blanching > blanching + osmosis > blanching + ultrasound > blanching + ultrasound assisted osmosis. However, the pore volume with a diameter below 0.5 μm showed an opposite trend. It was speculated that the immersion pretreatment led to volume shrinkage due to sample dehydration, and the penetration of maltodextrin penetrated into the material to occupy part of the space volume. Due to the cavitation effect of ultrasound pretreatment, the macropores of the sample shrunk and the number of small pores increased. Zhang et al. found that the microstructure of potato chips changed after ultrasound pretreatment, the pores with a diameter of 0.4–3 μm increased slightly, while the pores with diameters of 11–300 μm decreased (22). For samples subjected to different pretreatments followed by vacuum frying, the total pore volume, pore volumes with a diameter of 5–50 μm and 0.5–5 μm decreased, while the pore volume with a diameter above 50 μm did not change significantly, but the pore volume for diameters below 0.5 μm increased. The results are not the same as those of atmospheric fried potato chips (22).

**FIGURE 4:**
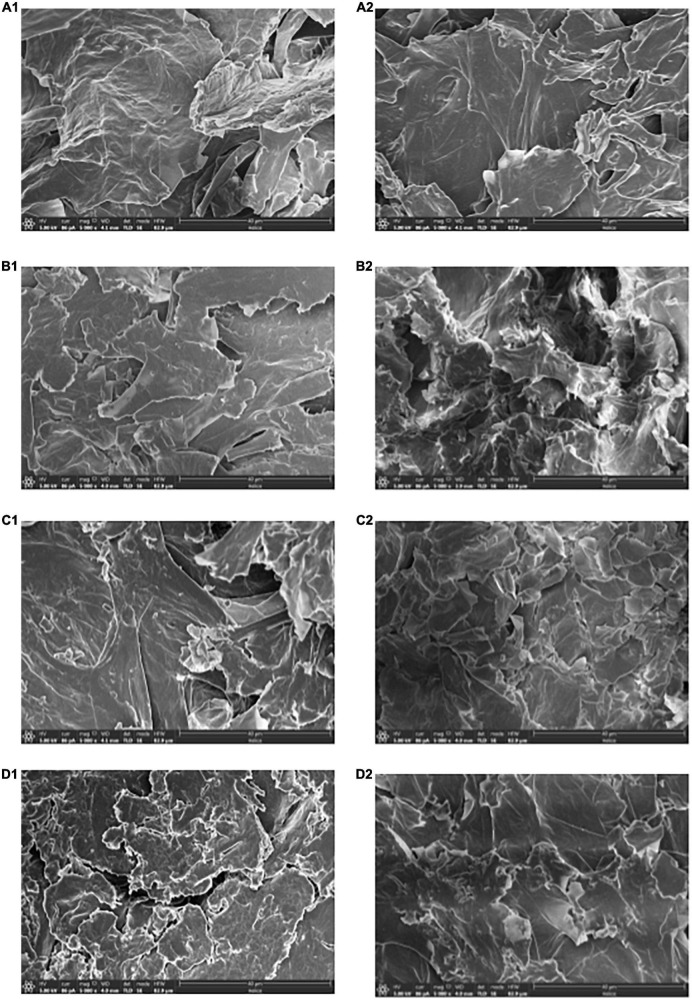
SEM images of *Pleurotus eryngii* chips before frying **(A1–D1)** and after frying **(A2–D2)**: **(A)** Blanching; **(B)** Blanching + osmosis; **(C)** Blanching + ultrasound; **(D)** Blanching + ultrasound + osmosis.

Figures 2, 3 showed that the pore diameter of *P. eryngii* was mainly distributed in the range of 5–50 μm, it was more appropriate to observe using SEM with a magnification of 5,000 times. As shown in Figure 4, the pore structure of *P. eryngii* chips with different pretreatments was in the following order from loose to tight: blanching, blanching + osmosis, blanching + ultrasound, blanching + ultrasound assisted osmosis, which was consistent with the results shown in Figures 2, 3. After vacuum frying (A2–D2), the number of pores in the four different pretreated *P. eryngii* chips was further significantly reduced compared to that before frying (A1–D1). Changes in pores with a diameter less than 0.5 μm were difficult to observe by SEM. Because osmosis causes pore shrinkage through osmotic dehydration and solute diffusion, Wang et al. (37) reported that osmotic dehydration could shrink the cell structure of yam slices, thereby reducing their porosity. Ultrasound could cause cell fragmentation under the effect of shearing effect, cavitation effect and collision effect, which may lead to the contraction of macropores and a reduction in the number of pores. Compared with ultrasound pretreatment, the structure of *P. eryngii* slices treated by ultrasound assisted osmosis were more compact with fewer pores. Piyalungka et al. (19) observed that both osmosis and ultrasound assisted osmosis reduced the oil content of vacuum fried pumpkin slices. SEM showed that the blanching pretreatment samples had largest and most pores, while the osmosis group had fewer pores; in addition, the ultrasound assisted osmosis pretreatment samples had the least amount of large pores but most small pores. These findings were consistent with the effect of pretreatment on the pore structure of *P. eryngii* chips in this experiment. The quantitative data of pore characteristics measured by MIP were further qualitatively verified to a certain extent by SEM analysis of *P. eryngii* chips.

The reduction in pore number of *P. eryngii* chips after vacuum frying was inconsistent with the results of studies on potato slices subjected to atmospheric pressure frying (23, 26). Llorca et al. (38) investigated the effect of frying on the microstructure of frozen squid rings. The results revealed that the fibers of squid muscle tissue became closer after frying. During the frying process, water vapored at high temperature, and migrated from the center of the sample to the outer layer, resulting in the changes of microstructure and formation of microchannels during the frying process. Pore characteristics are closely related to raw material composition, processing methods and other factors. It may also be due to the different structures of raw materials. For example, the structure of *P. eryngii* is spongy, while the structure of potato chips is relatively close.

### Quality analysis of vacuum fried *Pleurotus eryngii* chips

To compare the effects of different pretreatments on the quality of vacuum-fried *P. eryngii* chips, the physicochemical properties such as hardness, rehydration ratio, contents of reducing sugar and protein were investigated. As shown in Table 1, the contents of reducing sugar, protein, moisture and fat for fresh *P. eryngii* were consistent with previous study (1, 39). Vacuum frying led to a decrease in the contents of reducing sugar, protein and moisture and an increase in fat content, which was attributed to the loss of water – soluble during blanching process and fat-soluble nutrients during the vacuum frying process. This result was confirmed by Lu et al., who found that there existed many nutrients, such as polysaccharides, soluble proteins, and soluble solids in the blanching water of *P. eryngii* (40). Furthermore, vacuum frying process resulted in increased oil content and loss of fat-soluble nutrients in samples (41). There were no significant differences in reducing sugar, protein and moisture contents of vacuum fried *P. eryngii* chips with different pretreatments. Hardness could be used to measure the crispness of *P. eryngii* chips, the lower hardness indicated the higher crispness (42), therefore, the crispness of different pretreatments from high to low was blanching + ultrasound assisted osmosis > blanching + ultrasound > blanching + osmosis > blanching, this may be due to immersion of maltodextrin increase the crispness of the *P. eryngii* chips, and the change of structure caused of samples by ultrasonic pretreatment also increased the crispness of the *P. eryngii* chips. The results of rehydration rate were consistent with the crispness, while opposite with oil content. This may be due to ultrasound changed the intercellular space of *P. eryngii* chips, and microporous pores was formed (20), which caused the increased rehydration rate of *P. eryngii* chips. The reduction of oil content was in agreement with what was obtained by obvious research (24). The mechanism of ultrasonic assisted osmosis reduction in oil absorption was due to ultrasound pretreatment enhanced the movement of moisture within the structure of samples during vacuum frying. This created high vapour pressure within the structure of samples which then lowered the absorption of oil during frying (24). Moreover, when the chips were deoiled, changes in organizational structure facilitate the discharge of oil, thereby reducing the oil content of the chips (20).

The color values of fresh and vacuum fried *P. eryngii* chips with different pretreatments were shown in Table 2. Except blanching + ultrasound assisted osmosis, all vacuum fried samples exhibited lower lightness (L* value) compared with fresh *P. eryngii*, while there were no significant difference about redness (a* value) and yellowness (b* value). Generally, the smaller the total color difference (Δ*E*), the closer the color to the fresh sample. As expected, the L* values closer to the fresh sample and lower Δ*E* values were found in blanching + ultrasound assisted osmosis and blanching + ultrasound samples (Table 2), indicating that the color parameters of these pretreatments were closer to those of fresh sample. This may be due to the fact that ultrasonic pretreatment destroyed the cell structure, which leaded to the outflow of polyphenols, preventing non-enzymatic browning reactions (including Maillard reaction, caramelization and chemical oxidation), thereby reducing color deterioration (20).

**TABLE 2:** The color analysis of *Pleurotus eryngii* chips.

	L*	a*	b*	△*E*
Fresh	76.27 ± 1.25^b^	2.03 ± 0.25^a^	21.11 ± 1.02^a^	
Blanching (Fried)	70.23 ± 1.38^d^	2.28 ± 0.22^a^	20.85 ± 0.95^a^	6.05 ± 0.85^a^
Blanching + Osmosis (Fried)	72.25 ± 1.72^d^	2.15 ± 0.25^a^	19.25 ± 1.14^a^	4.43 ± 0.55^b^
Blanching + Ultrasound (Fried)	75.55 ± 1.45^c^	2.06 ± 0.23^a^	19.62 ± 1.26^a^	1.66 ± 0.53^c^
Blanching + Ultrasound + Osmosis (Fried)	78.17 ± 1.32^a^	2.15 ± 0.21^a^	20.5 ± 0.95^a^	1.99 ± 0.42^c^

Values are displayed as mean ± standard deviation. Different letters in the same column indicate significant differences (*P* < 0.05).

Overall, the *P. eryngii* chips treated by blanching + ultrasound assisted osmosis showed the best quality in terms of crispness, rehydration rate, oil content and color. Ultrasound assisted osmosis could be used to produce high-quality vacuum fried *P. eryngii* chips with outstanding advantages in terms of controlling oil content.

**FIGURE 5:**
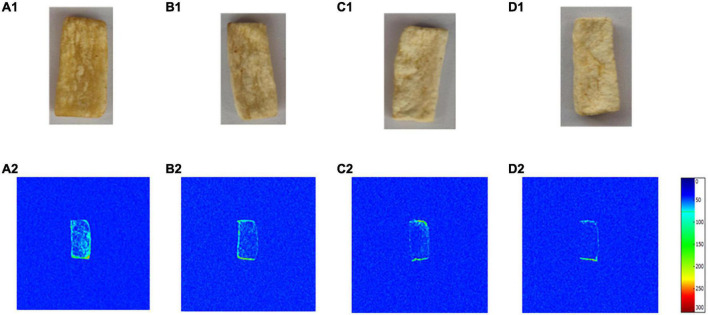
*Pleurotus eryngii* chips images **(A1–D1)** and LF-NMR images **(A2–D2)**: **(A)** Blanching; **(B)** Blanching + osmosis; **(C)** Blanching + ultrasound; **(D)** Blanching + ultrasound + osmosis.

Figure 5 A1–D1 showed the product picture of vacuum fried *P. eryngii* chips with different pretreatments obtained by a digital camera, and A2–D2 showed the pseudocolor images of *P. eryngii* chips with different pretreatments obtained by LF-NMR imaging. LF-NMR images can present visual information on the distribution of the oil. The color difference indicated the intensity of the signal in the LF-NMR image, which was proportional to the hydrogen proton density (43). The color bars in Figure 5 from blue to red represented the increase of signal density. It could be observed that there was a significant difference in signal intensity among samples with different pretreatments. The oil content in the samples gradually decreased from A to D (Figure 5). The results were consistent with the oil content of samples in Table 1. Meanwhile, A2–D2 in Figure 5 show that the signal at the edge of *P. eryngii* chips was stronger than that of other areas, indicating the oil content in the edge area of the vacuum fried sample was higher than in the other areas. It was similar to the findings reported by Isik et al. and Wang et al. (44, 45). It was speculated that the explanation for the variation in oil distribution was that, during the vacuum frying process, the oil was directly in contact with the edge areas, where it first adsorbed before gradually penetrating into the central of the sample. In addition, a large number of pores and cracks were formed on the surface of the samples due to the evaporation of water. They were not only the channels for water evaporation, but also for the penetration of oil into the interior of the sample. Furthermore, surface roughness played a crucial role in the permeability of oil (23).

### Relationship between pore structure and oil content

The difference in oil content of vacuum fried *P. eryngii* chips with four pretreatment as presented in Table 1 and Figure 5 may be caused by the different pore characteristic. Pearson correlation between oil content and pore size distribution of vacuum fried *P. eryngii* chips was investigated and results were shown in Table 3. The oil content of vacuum fried *P. eryngii* chips was significantly and positively correlated with the pores with diameters in the range >50, 5–50, and 0.5–5 μm of the samples both before and after vacuum frying, while the oil content showed a negative correlation with the pores with diameters below 0.5 μm. Vauvre et al. (46) considered that the more pores with pore size of 10–200 μm resulted in the higher oil permeability. Zhang et al. (23) believed that oil content was significantly positively correlated with pores with diameters of 100–200 and 10–100 μm, and negatively correlated with pores with diameters of 0.3–10 μm. Dueik et al. also believed that oil may not penetrate into micropores (<1.25 nm) (34). These conclusions were consistent with this experiment.

**TABLE 3:** Numerical values of Pearson’s correlation coefficients between oil contents and pore structure.

Pore structure			Pearson correlation coefficient	Significance difference
			*R*	*p*
Before frying	Pore diameter (μm)	>50	0.9977	0.0023
		5–50	0.9984	0.0016
		0.5–5	0.9952	0.0048
		<0.5	–0.9885	0.0116
After frying	Pore diameter (μm)	>50	0.9777	0.0223
		5–50	0.9957	0.0043
		0.5–5	0.9718	0.0282
		<0.5	–0.9823	0.0177

In the process of vacuum frying, water escapes from the surface of the samples and vaporizes violently to water vapor, thus pores are formed. The heated oil then penetrates into the samples through the pores. Heated oil is adsorbed on the surface of the samples and then penetrates inward through the pores, so oil absorption was related to pore structure (34). The oil content of the product was significantly negatively correlated with the pores with diameters below 0.5 μm. It was attributed that some of the pores were not connected to the surface or a possible force prevented oil to migrate deeper into products (22). Besides, the effects of pore on oil absorption differ from pore size, more amount of the pores with diameters above 0.5 μm led to more oil penetration. The increase in the number of pores with bigger diameters created less tortuous channels for oil penetration (47). In summary, ultrasound assisted osmosis pretreatment induced the formation of less macropores and more micropores than the other pretreatments, which prevented oil penetrates into the samples.

## Conclusion

The oil absorption of vacuum fried *P. eryngii* chips was affected by the porous structure. The oil content of vacuum fried *P. eryngii* chips was significantly and positively correlated with the pores with diameters above 50, 5–50, and 0.5–5 μm in the samples both before and after vacuum frying, while negatively correlated with the pores with diameters below 0.5 μm. Ultrasound pretreatment changed the microporous structure of *P. eryngii* chips, effectively hindering the oil absorption of samples. In particular, ultrasound assisted osmosis pretreatment induced the formation of more micropores. In summary, this manuscript revealed that the improvement of quality and the reduction of oil absorption of fried *P. eryngii* chips could be achieved by ultrasound assisted osmosis pretreatment before frying.

## Data availability statement

The raw data supporting the conclusions of this article will be made available by the authors, without undue reservation.

## Author contributions

XD and XM: conceptualization. AR, ZC, and XT: methodology. ZD: investigation. AR and XT: software. XD, XM, and AR: data curation. AR: writing – original draft preparation. AR and ZC: writing – review and editing. ZD, XM, and XD: supervision. XD, AR, and XT: funding acquisition. All authors contributed to the article and approved the submitted manuscript.
